# Suture-Based Vascular Closure Versus Surgical Closure of Large Bore Arteriotomies: A Real-World Experience

**DOI:** 10.7759/cureus.54856

**Published:** 2024-02-25

**Authors:** Keshava Murthy, Ratheesh Kumar J, Navjyot Kaur, Amitoj Chadha, Rajeev Chauhan, Davinder Chadha

**Affiliations:** 1 Cardiology Department, Army Hospital Research and Referral, New Delhi, IND; 2 Cardiology Department, Command Hospital Air Force, Bengaluru, IND; 3 Emergency Department, St. John’s Medical College, Bengaluru, IND; 4 Cardiology Department, Manipal Hospital, Bengaluru, IND

**Keywords:** vascular complications, technical success, open surgical closure, perclose proglide, preclose technique, large bore arteriotomies

## Abstract

Introduction: With the introduction of transcatheter aortic valve implantation (TAVI), endovascular abdominal aortic aneurysm repair (EVAR), thoracic endovascular aortic aneurysm repair (TEVAR), and frequent use of left ventricular assist devices in complicated percutaneous coronary interventions, the use of large bore arterial access has become a necessity. In the index study, we compared the percutaneous closure of large arteriotomies with open surgical (OS) closure.

Methods: It was a prospective study in which we compared the technical success and vascular complication rate associated with the use of a suture-based vascular closure device (VCD): Perclose ProGlide (PP) with that of OS closure. The study was carried out at Command Hospital Air Force, Bengaluru, India, from January 1, 2016, to December 31, 2020. The inclusion criteria were any percutaneous intervention involving large bore arterial access (≥12 French (F) sheath). The exclusion criteria were any condition where a persistent need for vascular access at the end of the procedure was required. We noted the baseline characteristics and type of anesthesia for all patients. The primary outcome was technical success and major vascular complications, which included major local site bleeding: Bleeding Academic Research Consortium (BARC) 3 or more, failed hemostasis requiring a second intervention, and acute vessel occlusion. Total time taken for the procedure (TTP), time to ambulation (TTA), and time to discharge post-procedure (TTD) were noted for each patient. The secondary outcomes were any bleeding other than major, local hematoma sized >5 cm at 24 hours, pseudo aneurysm formation at 30 days, and acute limb ischemia at 30 days.

Results: A total of 120 patients (PP: 60 (males: 54, females: 6), OS: 60 (males: 50, females: 10)) were included in this study. The mean age of patients was comparable in both groups (PP: 71.8 ± 9.62 years and OS: 71.0 ± 7.76 years, p-value: 0.63). Total large arteriotomies (mean size: 18.03F ± 3.34) closed were 184 (PP: 90, OS: 94). The procedures performed were EVAR: 64 (PP: 30, OS: 34), TAVI: 38 (PP: 21, OS: 17), and TEVAR: 18 (PP: 9, OS: 9). All patients in PP group received dual ProGlide with preclose technique. All TEVAR procedures (total arteriotomies: 18) required a vascular sheath of ≥ 24F. There was no statistical difference between the mean size of sheaths used in the two groups. The technical success (PP: 95.55%, OS: 97.87%, 95% CI: -5.78%-10.98%, p-value: 0.48) and rate of major complications were similar in both groups. Three patients in the PP group who had failed hemostasis with two ProGlides were successfully managed with one additional Angioseal (6F) each. The occurrence of hematoma sized larger than 5 cm was significantly more in the PP group compared to the OS group (PP: 7 (7.78%), OS: 0 (0%), p-value: 0.006). While GA was used for all patients who underwent vascular closure with OS, only eight patients (13.33%) in the PP group required GA. The TTP, TTA, and TTD were significantly lower in the PP group as compared to the OS group.

Conclusion: The percutaneous closure of large bore arteriotomies with suture-based VCDs is equally effective and is not associated with increased major vascular complications. In fact, the TTP, TTA, and TTD are significantly lower in the PP group which can translate to better patient comfort and lower costs.

## Introduction

With the advent and evolution of transcatheter aortic valve implantation (TAVI), endovascular abdominal aortic aneurysm repair (EVAR), and thoracic endovascular aortic aneurysm repair (TEVAR), large bore arteriotomies are a norm. As the population ages, many patients with prohibitive surgical risk require high-risk percutaneous coronary interventions (PCI) with the assistance of left ventricular assist devices (LVAD) like Impella. These devices also require large-bore arterial access. Initially, all these large bore accesses required surgical exposure of femoral/iliac arteries and their subsequent surgical closure. The involvement of a vascular surgeon requires prolonged anesthesia (often general anesthesia (GA)), extra procedural time, and prolonged post-procedure recovery. Not only this, but with surgical exposure, the local site wound is bigger and the chances of infection are greater. The wider use of vascular closure devices (VCDs) has made the percutaneous closure of these large arteriotomies easier. Non-surgical access and closure of large vascular accesses decrease the procedure time, reduce the requirement of GA, and augment the early ambulation and discharge of patients.

Initial observational studies demonstrated the safety of suture-based VCDs for large bore venous access sites. In an observational study involving 343 patients undergoing structural heart disease interventions with a mean venous sheath diameter of 11.5 ± 3 French (F), the use of suture-based VCDs with a preclose technique was found to be safe and effective [1]. These devices were also found to be safe and useful for closing large arteriotomies (mean arterial sheath diameter of 13 ± 2F); immediate hemostasis was achieved in 96% of cases [2]. A meta-analysis published in 2017 found that VCDs are comparable to surgical closure during transcatheter aortic valve repair (TAVR), EVAR, and TEVAR [3]. A decade-old literature quoted a vascular complication rate of 10-20% after TAVR [4,5]; however, the recent literature shows a vascular complication rate between 2.8% and 3.8% [6,7]. The reduction in vascular complication rates has been attributed to decreased sheath size with better delivery systems and improved skills of the operators. A better patient selection has also added to the reduction in vascular access site complications [8].

In the index retrospective study, we compared the vascular complications after the closure of large-bore arteriotomies using suture-based VCDs and surgical closure. For large bore access, two VCDs are approved: Perclose ProGlide (Abbott Vascular Inc., Santa Clara, USA) and Prostar XL (Abbott Vascular Inc., Santa Clara, USA). For large bore access, the preclose technique is used where the sutures are placed prior to the procedure; however, the knot is deployed afterward [9]. In our study, VCD Perclose ProGlide (PP) was used with the preclose technique, wherein two devices were pre-deployed at 10 and 2 o’clock positions prior to the procedure.

## Materials and methods

It was a prospective study in which we compared the technical success and complication rate associated with the use of PP closure for large bore arteriotomies with open surgical (OS) closure. The Institutional Ethics Committee of Command Hospital Air Force (CHAF-IEC) issued approval 147/15. The study was carried out at Command Hospital Air Force, Bengaluru, India, from January 1, 2016, to December 31, 2020. The inclusion criteria were any percutaneous intervention involving large bore arterial access (minimum 12F sheath). In patients with PP closure, all vascular access was taken either with fluoroscopic guidance or using ultrasound. The exclusion criteria were any procedural/vascular condition where a persistent need for vascular access at the end of the procedure was required.

The baseline clinical and vascular access characteristics were noted in all patients. The primary outcome was technical success and major complications which included major local site bleeding: Bleeding Academic Research Consortium (BARC) 3 or more, failed hemostasis requiring a second intervention, and acute vessel occlusion. Technical success was defined as when hemostasis was achieved without any additional intervention. The time for surgical cut-down and closure was noted for all procedures requiring OS. The surgical cut-down time was noted from the start of the skin incision to securing the arterial access for the procedure. The surgical closure time was noted from the time the main procedure was finished and the vascular cut-down and surgical site were closed. The time for placing VCD started from taking the fluoroscopic/ultrasound-guided puncture to placing two ProGlides using the preclose technique. The time for closing of the puncture site was measured from the time the main procedure was finished to the time the ProGlides were successfully deployed. The type of anesthesia used and the total time for each procedure were noted. The total time taken for the procedure (TTP) was taken from the time the patient entered the catheterization laboratory to the time the patient was taken out of same. Total time to ambulation (TTA) and time to discharge post-procedure (TTD) were noted for each patient. The secondary outcomes were any bleeding other than major, local hematoma sized >5 cm at 24 hours, pseudo-aneurysm formation at 30 days, and acute limb ischemia at 30 days.

The outcomes were assessed as per standard statistical methods. All quantitative or continuous variables were estimated using measures of central tendency (mean, median) and measures of central dispersion (standard deviation). Qualitative or categorical variables were described as frequencies and proportions.

## Results

A total of 120 patients were included in our study: 60 (males: 54, females: 6) in the PP group and 60 (males: 50, females: 10) in the OS group. The procedures performed were EVAR-64 (PP: 30, OS: 34), TAVI-38 (PP: 21, OS: 17), and TEVAR-18 (PP: 9, OS: 9). All patients in the PP group received dual PP devices with preclose technique. Total large arteriotomies closed were 184 (PP: 90, OS: 94). The mean age of patients was comparable in both groups (PP: 71.8 ± 9.62 years and OS: 71.0 ± 7.76 years, p-value: 0.63). The primary access was the right common femoral artery in 106/120 patients (PP: 46, OS: 60). All TEVAR procedures (total arteriotomies: 18) required a vascular sheath of ≥ 24F. The mean size of sheaths used in large arteriotomies was 18.03F ± 3.34 and the median was 18F (14-25F). There was no statistical difference between the mean size of sheaths used in the two groups (PP: 17.75F (± 3.74) and OS: 18.14F (± 3.23), 95% CI: -0.8796-1.6481, p-value: 0.55). The use of 12-14F, 15-18F, and more than 18 sheaths were comparable in the two groups as the rest of the baseline clinical characteristics (Table 1).

**Table 1:** Baseline characteristics of cohort F: French; GA: General anesthesia

S. No.	Characteristics	Perclose ProGlide (PP) group (n=60)	Open surgical (OS) arteriotomy group (n=60)	p-value
	Mean age (years) mean ± SD	71.8 ± 9.62	71.0 ± 7.76	0.63
	Male, n (%)	56 (93.3%)	50 (83.3%)	0.15
	Diabetes, n (%)	20 (33.3%)	28 (46.7%)	0.27
	Hypertension, n (%)	18 (30%)	16 (26.7%)	0.79
	Coronary artery disease, n (%)	31 (51.7%)	26 (43.3%)	0.31
	Atrial fibrillation, n (%)	4 (6.7%)	9 (13.9%)	0.15
	Obesity, n (%)	15 (25%)	17 (28.3%)	0.99
	Smoking, n (%)	28 (46.7%)	35 (58.4%)	0.36
	Chronic kidney disease, n (%)	13 (21.7%)	13 (21.7%)	0.99
	Peripheral artery disease, n (%)	4 (6.7%)	2 (3.3%)	0.99
	Use of 12-14F sheath, n (%)	28 (31.11%)	22 (23.40%)	0.24
	Use of 15-18F sheath, n (%)	39 (43.33)	40 (42.55%)	0.92
	Use of >18F sheath, n (%)	23 (35.55%)	32 (34.04%)	0.83
	20F	6	11	
	21F	-	1	
	22F	4	10	
	24F	9	10	
	25F	4	-	
	Use of GA, n (%)	8 (13.33%)	60 (100%)	<0.0001

The technical success was similar in both groups (PP: 95.55%, OS: 97.87%, 95% CI: - 5.78%-10.98%, p-value: 0.48). The rate of major complications was also similar in the two groups (Table 2). Only one patient in the PP group who had acute vascular occlusion required surgical intervention. Three patients in the PP group who had failed hemostasis with two PP devices were successfully managed with one additional Angioseal (6F) each. The occurrence of hematoma sized larger than 5 cm was significantly more in the PP group compared to the OS group (PP: 7 (7.78%), OS: 0 (0%), p-value: 0.006) (Table 2). Sub-group analysis was carried out for various parameters. A 24-hour hematoma-sized >5 cm episode in the PP group was observed to be significantly related to the presence of hypertension (OR: 0.126, p-value: 0.022) and smoking (OR: 9.33, p-value: 0.046).

**Table 2:** Technical success and vascular complications associated with suture-based VCDs and open surgical closure of large arteriotomies BARC: Bleeding Academic Research Consortium; VCD: Vascular closure device

S. No.	Outcome	Perclose ProGlide (PP) group (n=90)	Open surgical (OS) arteriotomy group (n=94)	95% CI	p-value
	Primary outcome				
	Technical success	86 (95.55%)	92 (97.87%)	-5.78% to 10.98%	0.48
	Major complications				
	Major local site bleeding: BARC 3 or more	7 (7.7%)	5 (5.3%)	-5.22% to 10.39%	0.51
	Failed hemostasis requiring second intervention	3 (3.3%)	1 (1.06%)	-2.95% to 8.30%	0.29
	Acute vessel occlusion	1 (1.1%)	0 (0%)	-2.93% to 6.01%	0.31
	Secondary outcome				
	Minor bleeding at 24 hours	6 (6.67%)	3 (3.19%)	-3.31% to 10.91%	0.27
	Hematoma > 5 cm at 24 hours	7 (7.78%)	0 (0%)	2.20% to 15.19%	0.006
	Pseudo aneurysm at 30 days	2 (2.22%)	1 (1.66%)	-4.75% to 6.22%	0.78
	Acute limb ischemia at 30 days	1 (1.7%)	1 (1.7%)	-5.41% to 5.25%	1.00

While GA was used for all patients who underwent vascular closure with OS, only eight patients (13.33%) in the PP group required GA. The total time taken for vascular access and closure and TTP were significantly lower in patients who underwent arteriotomies’ closure with PP (Table 3). Likewise, the TTA and THP were significantly lower in the VCD group (Table 3). In PP groups the average time to first ambulation was 20.20 hours ± 2.18 compared to 38.30 hours ± 3.40 in the OS group (p<0.0001). The mean THP was also significantly less in the PP group (PP: 48.56 hours ± 8.40, OS: 98.46 hours ± 7.46 (p<0.0001)). At 30 days, secondary outcomes were not significantly different between the two groups. There were two patients in the OS group who developed access site infections compared to none in the PP group. Two patients (3.3%) developed pseudo aneurysms in the PP group at 30 days compared to only one patient (1.7%) in the OS group (p=0.61).

**Table 3:** Mean time taken for procedure, ambulation, and discharge to home in two groups EVAR: Endovascular abdominal aortic aneurysm repair; Min: Minute; TAVR: Transcatheter aortic valve repair; TEVAR: Thoracic endovascular aortic aneurysm repair; PP: Perclose ProGlide; OS: Open surgical

S. No.		Perclose ProGlide (PP) group	Open surgical (OS) arteriotomy group	Difference between means (95% CI for mean difference)	p-value
1	Mean time for procedure	TAVR (n=21): 92.57 min ± 22.40	TAVR (n=17): 136.35 min ± 57.15	43.78 min (38.1729 to 49.3871)	<0.0001
		TEVAR (n=9): 109.56 min ± 20.20	TEVAR (n=9): 169.58 min ± 45.58	30.02 min (15.21 to 44.82)	<0.0006
		EVAR (n=30): 105.53 min ± 17.56	EVAR (n=34): 130.41 min ± 9.15	84.88 min (78.00 to 91.76)	<0.0001
2	Mean time for vascular site access and closure	PP (n=90): 4.23 min ± 0.58	OS (n=94): 36.31 min ± 4.47	31.07 min (31.14 to 33.02)	<0.0001
3	Time for ambulation	20.20 hours ± 2.18	38.30 hours ± 3.40	18.10 hours (17.07 to 19.13)	<0.0001
4	Mean time to hospital discharge post procedure	48.56 hours ± 8.40	98.46 hours ± 7.46	49.58 hours (47.54 to 52.25)	<0.0001

## Discussion

Introduced in the 1990s, the VCDs aimed to improve patients’ comfort, and reduce time to hemostasis, TTA, and TTD. Initially, they were designed to replace the manual compression used to close the smaller arteriotomies. However, with increasing indications of TAVI, TEVAR, and EVAR, closing larger arteriotomies without surgical help became a much-felt need. The use of LVADs in high-risk PCIs made this need even more palpable. There are two types of devices approved for the closure of large arteriotomies: active approximators and passive approximators. Active VCDs physically close the arteriotomy sites and include suture-based VCDs: PP (Abbott Vascular Inc., Santa Clara, USA) and Prostar XL (Abbott Vascular Inc., Santa Clara, USA) [9,10]. Passive VCDs either deploy a plug, a sealant, or gel at the arteriotomy site and include Angio-seal (Terumo Interventional Systems, Somerset, USA) and collagen-based device MANTA TM (Essential Medical Inc., Malvern, USA) [10]. The preclose technique is used with PP and Prostar XL devices for closing large arteriotomies and involves more than one device per arteriotomy. In the preclose method, the sutures are placed before the procedure; however, the knot is deployed afterward [10,11]. The 10F Prostar XL, with four in-built sutures, has been successfully used for arteriotomies as large as 24F [11,12]; whereas the PP device (the preclose technique) has been approved for 12-21F arteriotomies [10,11]. With these devices, there is no intra/extra luminal implant material and immediate re-access is possible. However, there is a steep and long learning curve [13]. The passive VCD, MANTA is approved for closure of large arteriotomies up to 22F [9,10]. Angioseal is used as a “hybrid technique” along with suture-based VCDs for closing the large arteriotomies [14,15], as we used in three of our patients. Unlike the suture-based devices, the same access site cannot be used for 90 days in the case of Angioseal and 180 days in the case of MANTA [9,10].

Any local vascular/bleeding complication associated with cardiac interventions leads to significant morbidity and mortality [16]. Hence the VCDs should be able to achieve successful hemostasis without any increase in major complications. The technical success associated with VCDs in large bore access has been comparable to surgical closure [6-11,17,18]. There has been no increase in major vascular complications with the use of suture-based VCDs for closing large arteriotomies when compared to OS [6-11,17,18]. In our study as well, the technical success rate was >95%, which is comparable to most of the real-time registries. Unlike most of the studies, where the maximum size of the sheaths used was between 14F and 20F, in our cohort, 23 arteriotomies that were closed with VCDs were ≥ 20F (20F: 6, 22F: 4, 24F: 9, 25F: 4). With suture based VCDs, the wire is retained in vessel lumen even after deploying the device. In case of failed hemostasis, an additional suture-based/plug-based VCD can be used [15,16]. Hence the requirement of OS closure surgery has reduced for failed hemostasis. Three patients in our PP group (22F: 1, 24F: 1, 25F: 1) had failed hemostasis; which was managed with one extra Angioseal device (hybrid technique) [15,16]. In a recent trial, the clinically significant bleeding complication rate in closing the large bore access with suture-based devices was 6% [19], in our study it was 7.7%. In another study assessing the safety of VCDs for transfemoral TAVR, the use of PP was found to be safe and effective with only 3.2% minor bleeding, nil major vascular complications, and short hospital stay (3.1 ± 3.7 days) with a device failure rate of only 4.8% [20]. Both these studies had lesser bleeding complications compared to our study due to the use of relatively smaller vascular sheaths with newer valves. There are only a few studies available that assess the outcome of suture-based VCDs in very large vascular access (≥20F). In one such study, the average sheath size was 18.09 ± 1.55F, which showed device success of 98.4%, however, 4 patients (1.6%) developed critical stenosis of the femoral artery requiring intervention [21]. Though there has been an increase in the risk of acute vessel occlusion with plug-based VCDs, the risk is much lower with suture-based VCDs [22-25]. In our PP cohort, only one patient required surgery for the above indication. The experience of the operator is directly linked to device success and inversely related to vascular complications in PP [26]. Seven patients in the PP group had hematomas which were managed with compression which was significantly more in patients with uncontrolled hypertension. Three patients in the OS group had local infection which required prolonged antibiotics. The risk of infections and seromas has decreased with the use of VCDs [18,19,27]. Two patients in the PP group and one in the OS group had pseudo aneurysms which were again managed with local compression. One patient in the PP and OS group developed acute limb ischemia at 30 days, however, both patients had pre-existing atrial fibrillation.

One of the main advantages of percutaneous interventions over OS surgery is early ambulation and reduced hospital stay [18,19]; hence any intervention that promotes these parameters without hampering the efficacy of the primary procedure has the potential to augment the gains from the intervention. The time to achieve and close the vascular access and hence TTP is significantly higher in the OS group (PP: 4.23 min ± 0.57, OS: 36.31 ± 4.47, mean difference > 30 mins/arteriotomy, p-value<0.0001). Similarly, the TTA and THP are significantly lower in the PP group. While all patients in the OS group required GA, only a few (13.33%) required GA. With increasing age and co-morbidities, the number of high-risk PCIs that require LVADs like Impella has increased. The LVAD like Impella may require vascular access of size as large as 23F. The requirement of vascular surgeon and mandatory GA, negate the advantage of percutaneous management of prohibitive surgical risk patients as GA itself is associated with inherent risks, and not all cardiac patients may tolerate the same before they are placed on LVAD. Besides closing all large arteriotomies with OS would not only increase the TTP but also the total GA time.

Before we conclude the discussion, one important aspect is the economic impact of early discharge after the procedure. After the intervention, most of these patients stay in intensive care units for monitoring. In the index study, the patients who had vascular access closed using the PP approach were discharged at an average of almost 50 hours earlier than the patients who were managed with OS; irrespective of the procedure. An Indian study done at a tertiary care hospital in Northern India revealed that if patients are discharged same day (SDD) after PCI versus the next day discharge (NDD), an amount of INR 36,178.57 could be saved per SDD [28]. The mean time difference between SDD and NDD in that study was 13.05 hours. Taking the same calculation in the index cohort, with 50 hours of hospitalization curtailed, we would be able to save INR 1,38,615.21 per patient if they have a vascular closure using PP. Though doing a separate economic analysis for PP and OS in large bore arteriotomies would give a much more authentic figure, still the amount projected to be saved per patient by early discharge cannot be ignored. In the analysis mentioned above, the man-hours spent by anesthesiologists, vascular surgeons, and their team were not taken into account. Similarly, the cost of VCDs has not been considered.

Limitations

Our study has vital limitations. It was an observational study where the patients who were selected for PP and OS vascular closure were chosen by the primary operators and there was no randomization. A selection bias cannot be ruled out. The sample size in our study was rather small (total patients=120, total arteriotomies=184) and follow-up was only one month post-procedure. A longer follow-up would have brought out the late complications of large access arteriotomies.

## Conclusions

We conclude that suture-based VCDs are equally effective in closing the large bore arteriotomies without any increase in major vascular complications. With percutaneous vascular closure, the requirement of GA decreases considerably for transcatheter interventions and so does the TTP, TTA, and THP. The total percutaneous transcatheter procedures are also projected to reduce the total costs spent per procedure.
